# Brom-Ethyl as an Anæsthetic

**Published:** 1889-01

**Authors:** 


Brom-ethyl as an Anæsthetic.—Dr. Szuman recommends inhalations of brom-ethyl for short operations. The patients wake up rapidly and no vomiting or debility is produced. Medical aid is not required in its administration. It should not be used in protracted operations, or in drinkers, consumptives, and persons suffering from cardiac or renal disease. It is administered with the mask employed for chloroform anæsthesia.—Translated from *Therapeut. Monatshefte*, April and May, 1888

